# Integrative multi-omics and machine learning identify mechanical stimulus-related genes and immune microenvironment remodeling in heart failure

**DOI:** 10.3389/fcvm.2026.1953634

**Published:** 2026-10-28

**Authors:** Cheng Zhang, Linna Zhao, Jiahuan Sun, Xiaodan Wang, Chuang Zhang, Aiying Li

**Affiliations:** 1 Department of Biochemistry and Molecular Biology, College of Pharmacy, Hebei University of Chinese Medicine, Shijiazhuang, Hebei, China; 2 Transformation Center for Scientific and Technological Achievements, Hebei University of Chinese Medicine, Shijiazhuang, Hebei, China; 3 Hebei key Laboratory of Chinese Medicine Research on Cardio-Cerebrovascular disease, Hebei University of Chinese Medicine, Shijiazhuang, Hebei, China

**Keywords:** diagnostic biomarker, heart failure, immune microenvironment, machine learning, mechanical stimulus, single-cell transcriptomics

## Abstract

**Background:**

Heart failure (HF) is a leading cause of global morbidity and mortality, driven by complex pathophysiological processes including abnormal mechanical stress and immune microenvironment remodeling. Although mechanical stimulus is known to trigger maladaptive cardiac remodeling, the systematic role of mechanical stimulus-related genes (MSRGs) in HF diagnosis and immune modulation remains poorly defined. This study aims to identify MSRG-based diagnostic biomarkers, characterize molecular subtypes, and explore their cellular localization and immune microenvironment interactions in HF.

**Methods:**

Bulk transcriptomic data and MSigDB-curated MSRGs were analyzed. Differential expression screening identified MSRGs altered between HF and healthy specimens. Three algorithms—Random Forest, LASSO, and SVM-RFE—were jointly applied to select diagnostic biomarkers and build a nomogram. Consensus clustering defined MSRG-based subtypes, with immune profiles compared across groups. Single-cell sequencing localized key biomarkers in cardiac tissues. A transverse aortic constriction (TAC)-induced rat HF model served for *in vivo* validation via RT-qPCR, Western blotting, and immunohistochemistry.

**Results:**

A total of 53 MSRG-related DEGs were identified, from which 11 core diagnostic biomarkers were selected. The nomogram model showed high predictive performance. Consensus clustering revealed two distinct HF subtypes: Cluster A exhibited enhanced metabolic and homeostatic pathways, while Cluster B showed pronounced extracellular matrix remodeling and fibrotic features, with differential immune infiltration. Single-cell analysis demonstrated CASP1 enrichment in macrophages/monocytes and PTN/MDK enrichment in fibroblasts. *In vivo* validation confirmed significant upregulation of PTN, MDK, and CASP1 at both mRNA and protein levels in TAC hearts, alongside elevated pro-inflammatory cytokines (IL-6, IL-1β) and fibrotic markers, linking mechanical stimulus-related genes to local inflammation and adverse remodeling.

**Conclusion:**

MSRGs serve as robust diagnostic biomarkers for HF and enable the identification of two molecular subtypes with distinct immune microenvironment and biological pathway profiles. The CASP1-MDK-PTN axis emerges as a potential pathogenic cascade connecting mechanical stress to inflammatory and fibrotic remodeling. These findings provide a framework for early molecular subtyping, improved risk stratification, and targeted immunomodulatory therapy in HF.

## Introduction

Heart failure (HF) remains a leading cause of global morbidity and mortality, characterized by progressive ventricular remodeling and an irreversible decline in cardiac function (1–3). Despite significant advances in pharmacological and device therapies, the clinical management of HF is still hampered by its complex heterogeneity and the limitations of current diagnostic tools (4–6). Currently, clinicians frequently rely on N-terminal pro-BNP (NT-proBNP) and B-type natriuretic peptide (BNP) for prognostic and diagnostic purposes. However, these conventional markers frequently lack the sensitivity required for early-stage identification, accurate etiological sorting, and the formulation of individualized treatment plans (7–9). Consequently, there is an urgent clinical need to identify more robust, mechanism-driven diagnostic biomarkers that can capture the underlying pathophysiological complexity of HF and facilitate early, targeted intervention.

Molecular, neurohormonal, and hemodynamic shifts interact intricately to propel the pathophysiological evolution of HF, ultimately resulting in continuous pump failure and detrimental ventricular restructuring (10–12). Among these multifaceted drivers, abnormal mechanical stimulus—particularly chronic pressure overload induced by conditions such as systemic hypertension, aortic stenosis, or myocardial infarction—serves as a primary instigator of cardiac deterioration (13–15). Sustained mechanical stress initiates intricate mechanotransduction pathways that not only trigger cardiomyocyte hypertrophy and apoptosis but also profoundly activate resident cardiac fibroblasts and infiltrating immune cells (16–18). This cascade orchestrates a maladaptive immune microenvironment remodeling, characterized by chronic low-grade inflammation and excessive extracellular matrix (ECM) deposition (19–21). Although the pivotal role of mechanical stimulus in HF pathogenesis is well-established, how mechanical stimulus-related genes (MSRGs) systematically orchestrate disease progression and interact with the dynamic cardiac immune microenvironment remains largely unexplored. A comprehensive, system-level understanding of these gene-microenvironment interactions is critical for unraveling the intricate mechanisms of HF and discovering novel therapeutic targets.

Our research capitalized on extensive bulk transcriptome profiles combined with a meticulously compiled collection of MSRGs obtained from the Molecular Signatures Database (MSigDB). Utilizing a strict machine-learning framework that incorporated cross-validation, Support Vector Machine-Recursive Feature Elimination (SVM-RFE), Least Absolute Shrinkage and Selection Operator (LASSO) regression, and Random Forest (RF) algorithms, we isolated a highly reliable set comprising 11 essential diagnostic indicators. Beyond mere biomarker identification, we applied unsupervised consensus clustering to these signatures, unveiling two distinct molecular subtypes of HF. These subtypes exhibited divergent immune microenvironment profiles, with one cluster showing enhanced metabolic homeostasis and the other displaying a profound fibrotic and tissue-remodeling phenotype. We incorporated single-cell RNA sequencing (scRNA-seq) datasets to dissect this cellular diversity at an individual cell level. This sophisticated methodology enabled the exact spatial mapping of the identified markers. We discovered their distinct accumulation in macrophage-triggered inflammatory routes and fibroblast-led ECM restructuring, offering a novel perspective on intercellular communication in the compromised myocardium.

However, computational predictions and bioinformatic associations necessitate rigorous biological validation to confirm their translational relevance and causal roles. Therefore, we established a classic transverse aortic constriction (TAC)-induced rat model of HF to validate our findings *in vivo*, perfectly simulating the pressure-overload mechanical stress observed in human pathology. Through quantitative real-time PCR, Western blotting, and immunohistochemical staining, we confirmed the significant upregulation of key mechanical stimulus-related biomarkers, PTN, MDK, and CASP1, in the failing myocardium. Crucially, the spatial and molecular co-expression of these markers with established hallmarks of cardiac fibrosis and pro-inflammatory cytokines provided compelling *in vivo* evidence. This validation intrinsically links the dysregulation of mechanically driven genes to exacerbated local inflammation, immune microenvironment remodeling, and adverse cardiac deterioration. Collectively, this study not only identifies novel, mechanically driven diagnostic biomarkers with high predictive value but also provides a comprehensive, multi-omics atlas of immune microenvironment remodeling in HF. These findings offer a solid theoretical foundation for early molecular subtyping, improved risk stratification, and the future development of targeted immunomodulatory therapies in HF management.

## Materials and methods

### Data preprocess

Publicly accessible transcriptomic data formed the basis of this work. Specifically, two bulk RNA-seq datasets—GSE141910 (comprising 200 HF and 166 normal samples) (22), and GSE57338 (including 177 HF and 136 normal samples) (23) —were sourced from the GEO repository (http://www.ncbi.nlm.nih.gov/geo/). Platform specifications differed between the two: GSE141910 relied on the GPL16791 platform (Illumina HiSeq 2500 for human transcripts), while GSE57338 utilized the GPL11532 platform (Affymetrix Human Gene 1.1 ST Array). Because GSE141910 and GSE57338 were generated using different transcriptomic platforms, they were processed independently. Gene identifiers were mapped to common gene symbols, and for GSE57338, the probe with the highest mean expression was retained when multiple probes mapped to the same gene. No cross-platform batch correction was performed because the two datasets were analyzed separately rather than merged. Supplementary Table S1 lists the 228 mechanical stimulus-responsive genes that we isolated from the MSigDB v2025.1.Hs repository (GO:0009612, http://www.gseamsigdb.org/gsea/index.jsp). Moreover, single-cell transcriptomic profiles derived from seven transmural LV apex samples, including two donor and five HF samples, were obtained from GSE183852 (24). Individual cells were considered cellular observations nested within their respective biological samples and were not interpreted as independent biological replicates. These data were analyzed with the Seurat pipeline, and the cell-level filtration parameters were kept consistent with the initial study's descriptions.

### Differentially expressed gene analysis and functional analysis

To identify DEGs related to mechanical stimulation between HF and healthy samples within the GSE141910 cohort, we applied the limma R package (25). The statistical thresholds for screening were defined as a false discovery rate (FDR) below 0.05 and an absolute log2-transformed fold change (|log2FC|) exceeding 0.5. Subsequently, the DEGs linked to mechanical stimulation underwent functional enrichment examination through Gene Ontology (GO) terms and Kyoto Encyclopedia of Genes and Genomes (KEGG) pathways via the Metascape online tool (26), Statistical significance was defined as an adjusted *P*-value threshold below 0.05.

### Screening and validation of mechanical stimulus diagnostic biomarkers

For identifying novel and meaningful HF-related biomarkers, three separate machine learning algorithms were employed: RF, LASSO logistic regression, and SVM-RFE. Random Forest analysis utilized the “randomForest” package in R (27), whereas LASSO regression employed the “glmnet” package (28). RF analysis was performed with 500 trees, and variable importance was assessed using IncNodePurity; genes with IncNodePurity >1 were retained. LASSO logistic regression was performed using a binomial model with an L1 penalty (alpha = 1) and 50 lambda values. Five-fold cross-validation was used to select the optimal penalty parameter, and genes with non-zero coefficients at lambda.min were retained. SVM-RFE was performed using a radial-basis-function SVM with five-fold cross-validation, evaluating feature-set sizes from 1 to 53. Random seeds of 1, 2, and 7 were used for RF, LASSO, and SVM-RFE, respectively. The genes shared by all three feature-selection methods were retained as the final candidate feature panel. To further evaluate the reproducibility and diagnostic performance of the identified 11-gene panel in an independent cohort, the GSE57338 dataset was analyzed using the same predefined gene set. A logistic regression model was constructed based on these genes, and its discriminatory performance was assessed using receiver operating characteristic (ROC) curve analysis, with the area under the curve (AUC) serving as the quantitative metric. Calibration curves were generated to evaluate the agreement between predicted and observed probabilities, while decision curve analysis (DCA) was performed to assess the potential clinical net benefit of the model across a range of threshold probabilities.

### Identification of mechanical stimulus molecular subtypes

HF patients were categorized into separate molecular subtypes associated with mechanical stimulus through unsupervised clustering methodology, employing diagnostic biomarker expression profiles and the “ConsensusClusterPlus” R package (29). The clustering was performed with the K-means algorithm, adopting Euclidean distance as the similarity metric, and the robustness of the clustering was ensured by resampling 80% of the items across 1,000 iterations. The optimal number of clusters was determined by jointly evaluating the consensus matrix, cumulative distribution function (CDF), proportion of ambiguous clustering (PAC), and cluster consensus scores. PAC was calculated as the proportion of consensus values within the ambiguous interval of 0.1–0.9, with lower PAC values indicating greater clustering stability. Following this, a heatmap was generated to verify biomarker gene expression profiles among the discovered subtypes. Immune cell infiltration in the bulk transcriptomic data was estimated using the CIBERSORT deconvolution algorithm with the LM22 leukocyte signature matrix, which was used to estimate the relative proportions of 22 immune cell subsets in each sample. Differences in the estimated immune cell proportions and immune checkpoint gene expression between the two subtypes were evaluated using the Wilcoxon rank-sum test. Furthermore, the subtype-associated genes were subjected to GO and KEGG enrichment analyses via the Metascape database.

### scRNA-seq data analysis

Single-cell RNA-seq data from GSE183852 were analyzed using Seurat (30). The dataset comprised seven transmural left ventricular (LV) apex samples, including two donor and five HF samples. Cells with fewer than 300 or more than 5,000 detected genes, or with mitochondrial gene percentages >10%, were excluded. After quality control, the remaining cells were merged into a single gene-expression matrix, followed by normalization and scaling using NormalizeData() and ScaleData(). Dimensionality reduction and clustering were performed using UMAP and Seurat clustering functions. Cell-type annotations provided with the original dataset were retained. Individual cells were considered observations nested within their respective biological samples rather than independent biological replicates.

Candidate gene expression was examined across cell types and between donor and HF groups. For cell-type-specific functional analysis, monocytes/macrophages and fibroblasts were analyzed separately and divided into high- and low-expression groups according to the median expression of the corresponding candidate gene. Differentially expressed genes were identified using the Wilcoxon rank-sum test with a log2 fold-change threshold of 0.5. Genes with an adjusted *P* < 0.05 and average log2 fold change >0.5 were subjected to GO Biological Process enrichment analysis using clusterProfiler and org.Hs.eg.db (31).

Cell–cell communication was inferred using CellChat with the “Secreted Signaling” subset of the human CellChatDB database. Overexpressed genes and ligand–receptor interactions were identified and projected onto the human protein–protein interaction network. Communication probabilities were calculated, and interactions involving cell populations with fewer than 10 cells were excluded. Pathway-level communication was subsequently calculated and aggregated to characterize the number and strength of interactions among cell populations.

Pseudotime analysis of monocytes and macrophages was performed using Monocle3. Following normalization, variable-feature identification, scaling, PCA, and UMAP using the first 20 principal components, cells were converted into a Monocle3 cell_data_set for trajectory reconstruction. Monocytes were designated as the root population, and cells were ordered along the inferred pseudotemporal trajectory.

### CeRNA-network construction

For exploring post-transcriptional regulation mechanisms, a competing endogenous RNA (ceRNA) network was established. Transcription factors (TFs) represent regulatory proteins capable of recognizing and attaching to specific DNA sequences, thus regulating target gene transcription. Candidate gene-TF interactions were examined via the NetworkAnalyst 3.0 web platform (https://www.networkanalyst.ca/) (32). Simultaneously, microRNAs (miRNAs) represent endogenous small non-coding RNA molecules that mainly function through enhancing mRNA degradation or inhibiting target transcript translation. The same set of common genes was subsequently submitted to NetworkAnalyst 3.0 to construct a comprehensive regulatory network linking these genes with their putative miRNA interactors. The resulting ceRNA network represents computationally predicted regulatory relationships and should not be interpreted as experimentally validated molecular interactions.

### Establishment of the TAC animal model

Male Sprague-Dawley rats (150–170 g) from Beijing Charles River Laboratory Animal Technology Co., Ltd. were housed under controlled conditions (23 °C, 55%–60% humidity) with free access to food and water for 7 days prior to surgery. Pressure-overload HF was induced by TAC under intraperitoneal anesthesia with 0.3% pentobarbital sodium. A 27-gauge needle and 6-0 silk suture were placed around the transverse aorta for ligation according to standard protocols. Sham animals underwent identical operations without aortic constriction. At 4 weeks post-surgery, echocardiography confirmed HF phenotype establishment. Rats were randomly assigned to experimental groups (initially *n* = 6 per group to account for potential surgical mortality associated with the TAC procedure). For downstream molecular analyses, including RT-qPCR and Western blotting, tissues from 3 successfully modeled rats per group were randomly selected and utilized. Thus, all molecular assays were performed with *n* = 3 independent biological replicates (three distinct animals), rather than technical replicates. Of note, although part of these tissue samples had been used in our previous report (33), the present work repurposed this cohort to investigate novel mechanisms underlying HF pathogenesis.

### Western blot analysis

Rat left ventricular myocardial tissues were lysed using lysis buffer. Equal amounts of protein were separated by SDS-PAGE, then transferred onto PVDF membrane and sealed at room temperature for 2 h with 5% skim milk diluted with TBST. Subsequently, the primary antibody was incubated overnight at 4 °C. The employed antibodies encompassed anti-NPPA (rabbit polyclonal, 1:1,000, 27426-1-AP, Proteintech), anti-BNP (rabbit polyclonal, 1:1,000, PA5-96084, Invitrogen), anti-β-MHC (rabbit monoclonal, 1:2,000, 85322-1-RR, Proteintech), anti-Collagen I (rabbit polyclonal, 1:2,000, PA1-26204, Invitrogen), anti-Collagen III (rabbit polyclonal, 1:2,000, 22734-1-AP, Proteintech), anti-α-SMA (rabbit polyclonal, 1:2,000, 14395-1-AP, Proteintech), anti-Caspase-1 (rabbit polyclonal, 1:1,000, 31020-1-AP, Proteintech), anti-Pleiotrophin (rabbit polyclonal, 1:1,000, PA5-94984, Invitrogen), anti-Midkine (rabbit polyclonal, 1:1,000, PA5-115560, Invitrogen), and anti-GAPDH (rabbit polyclonal, 1:5,000, 10494-1-AP, Proteintech). Subsequently, the membranes were incubated with the appropriate horseradish peroxidase -conjugated secondary antibodies for 90 min at room temperature. Immunoreactive signals were finally developed using an enhanced chemiluminescence substrate kit and captured with an OmegaLum W chemiluminescent imaging system (Minneapolis, MN, USA).

### Reverse transcription quantitative PCR (RT-qPCR)

We isolated total RNA from the left ventricular myocardium of rats utilizing the Omega Total RNA Kit II, strictly adhering to the guidelines provided by the manufacturer. A spectrophotometer was employed to measure the concentration of the extracted RNA. Subsequently, the MonScript™ RT III All-in-One Mix containing dsDNase was applied for cDNA synthesis. Gene expression profiling for Collagen I, α-SMA, PTN, PPL, Collagen III, STRBP, MDK, SLC2A1, CASP1, SERPINE2, BNP, GADD45A, PDE2A, ANP, β-MHC, and THBS1 was conducted via quantitative PCR utilizing the MonAmp™ ChemoHS qPCR Mix, normalizing the results against GAPDH as the endogenous control. Every quantitative PCR assay was executed in three independent replicates. The 2^−*ΔΔ*Ct^ approach was adopted to compute the relative abundance of transcripts. Detailed information regarding the primer sequences can be found in Supplementary Table 3.

### Immunohistochemical staining

Protein expressions of IL-6, IL-1beta, PTN, MDK and CASP1 were detected through immunohistochemical staining following PV-9,000 kit protocol (OriGene, Delaware, USA). Heart tissue specimens underwent paraffin embedding and were sectioned at 5 μm thickness. The tissue slices were dewaxed in xylene, rehydrated through a descending ethanol gradient, and subjected to antigen retrieval in a 0.01 mol/L citric acid buffer (pH 6.0) at 100 °C for a duration of 15 min. Endogenous peroxidase was quenched by incubating the slides in 3% H_2_O_2_, after which non-specific binding was blocked using 10% goat serum for half an hour. The tissue sections were then incubated with primary antibodies against Caspase 1 (1:200, rabbit polyclonal, 31020-1-AP, Proteintech), Pleiotrophin (1:200, rabbit polyclonal, PA5-94984, Invitrogen), Midkine (1:200, rabbit polyclonal, PA5-115560, Invitrogen), IL-1beta (1:200, rabbit polyclonal, 26048-1-AP, Proteintech), and IL-6 (1:200, rabbit polyclonal, GB11117-100, Servicebio) overnight at 4 °C. After incubation with secondary antibodies for 30 min at room temperature, the sections were rinsed with PBS and developed with DAB. Finally, images were captured using an optical microscope (Leica DM4000B, Leica Microsystems GmbH, Wetzlar, Germany).

### Statistical analysis

Statistical analyses were performed using R (version 4.1.1) and GraphPad Prism (version 9.0). For bulk transcriptomics, differential expression was analyzed via the *limma* package (FDR < 0.05, |log2FC| > 0.5). Single-cell comparisons utilized the Wilcoxon rank-sum test. For experimental validations (RT-qPCR, Western blotting, IHC), data are presented as mean ± SD or SEM. Group comparisons employed Student's *t*-test, one-way ANOVA, or Mann–Whitney *U* test, as appropriate. All molecular assays included at least three biological replicates (*n* ≥ 3). Statistical significance was defined as *P* < 0.05. Statistical significance was denoted as ns (*P* ≥ 0.05), *P < 0.05, **P < 0.01, ***P < 0.001, and ****P < 0.0001.

## Result

### Transcriptomic landscape of MSRGs in HF patients

Figure 1 depicts the comprehensive analytical pipeline utilized in this study. By comparing mechanical stimulus-associated transcripts in GSE141910 samples (HF vs. normal), we identified 53 differentially expressed genes (DEGs), which are visualized in the volcano plot of Figure 2A. Figure 2B displays a heatmap detailing the expression patterns for all 53 identified DEGs across the entire sample set. We generated a protein-protein interaction (PPI) network to investigate the functional interplay between these genes, as shown in Figure 2C. As illustrated in Figure 2D, Gene Ontology (GO) and Kyoto Encyclopedia of Genes and Genomes (KEGG) enrichment assessments demonstrated that these DEGs are primarily involved in biological processes such as cellular response to mechanical stimulus, sensory perception of mechanical stimulus, and response to mechanical stimulus. Additionally, they were associated with molecular functions like protease binding, and cellular components encompassing the collagen-containing extracellular matrix and the external side of the plasma membrane. Furthermore, the PI3K-Akt and HIF-1 signaling pathways were significantly enriched. To pinpoint diagnostic biomarkers pertinent to HF, we utilized three distinct machine learning techniques. Specifically, the SVM-RFE method picked 40 potential genes (Figure 2E), while the Random Forest (RF) approach identified 15 genes (Figure 2F), and LASSO regression analysis resulted in 27 genes (Figure 2G). The intersection of these three gene sets was visualized using a Venn diagram (Figure 2H), which ultimately uncovered 11 robust core biomarkers: PTN, PPL, STRBP, MDK, SLC2A1, CASP1, SERPINE2, NPPA, GADD45A, PDE2A, and THBS1.

**Figure 1:**
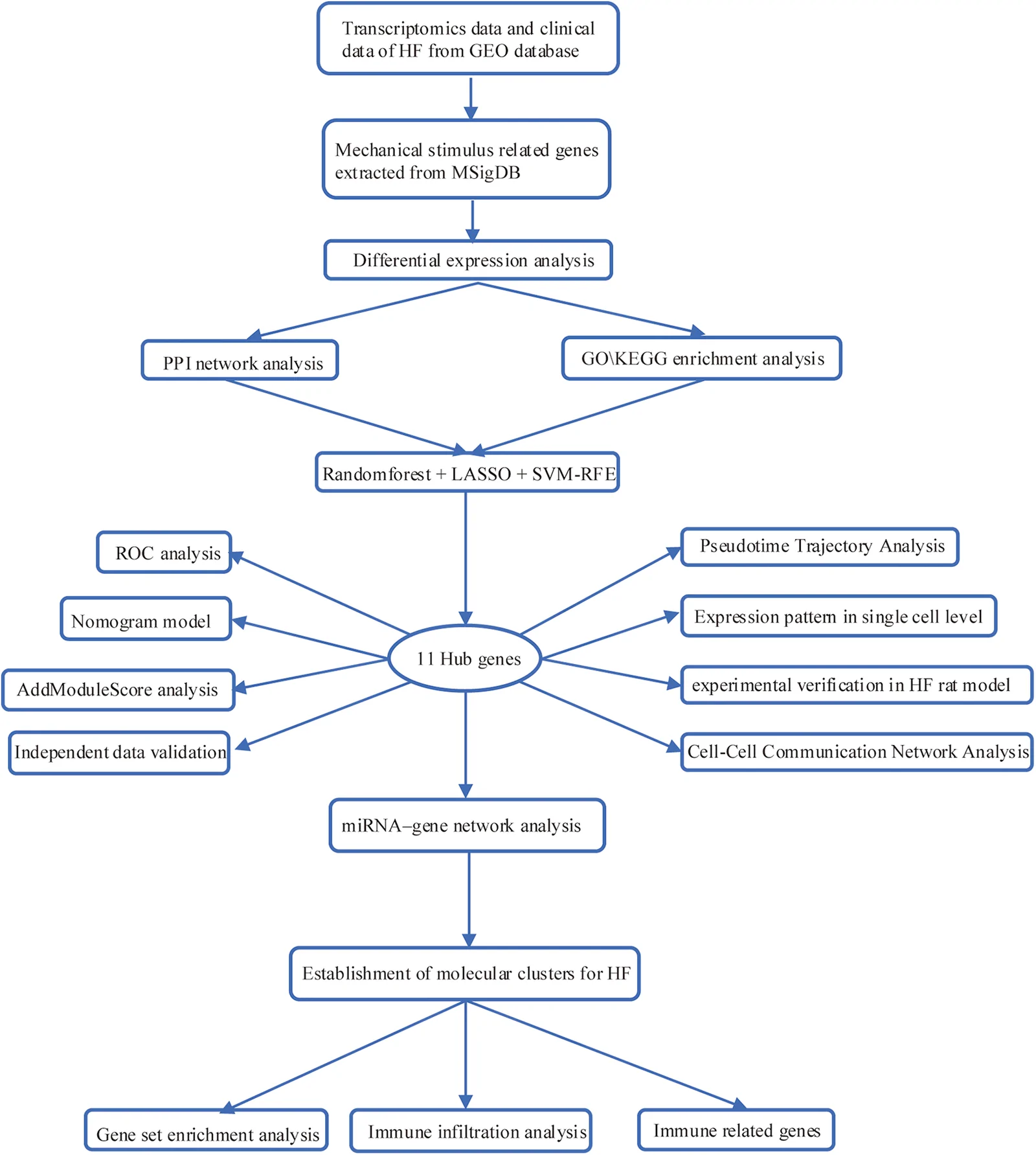
Schematic overview of the study workflow.

**Figure 2:**
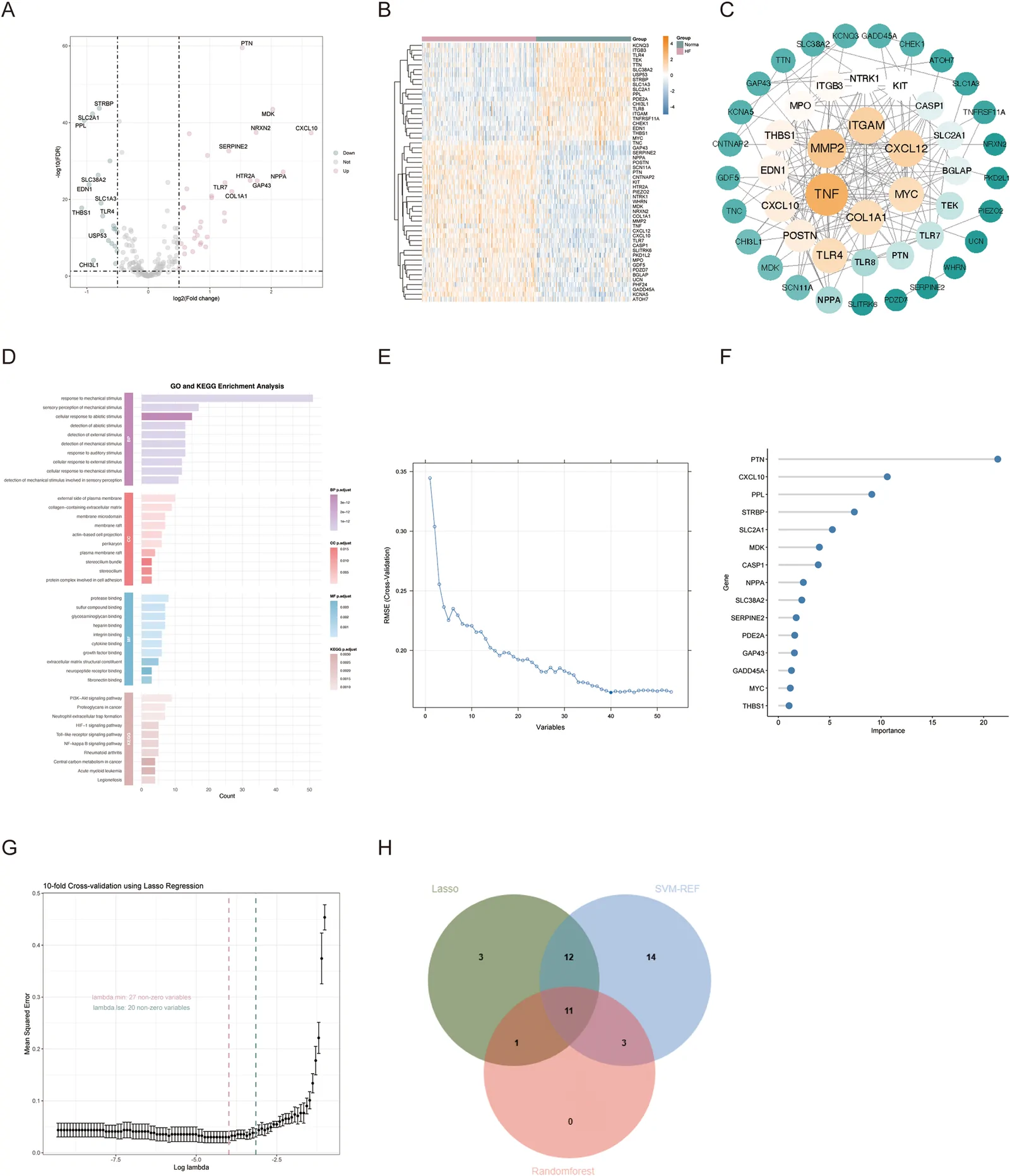
Identification of mechanical stimulus–related genes and screening of diagnostic biomarkers in heart failure. **(A)** Volcano plot showing differentially expressed mechanical stimulus–related genes (DEGs) between HF and healthy control samples in the GSE141910 cohort. **(B)** Heatmap displaying the expression patterns of the identified mechanical stimulus–related DEGs. **(C)** Protein–protein interaction (PPI) network of mechanical stimulus–related DEGs. **(D)** GO and KEGG enrichment analyses of the identified DEGs. **(E)** Feature selection using the SVM-RFE algorithm. **(F)** Variable importance ranking generated by the random forest (RF) model. **(G)** Least absolute shrinkage and selection operator (LASSO) regression analysis for biomarker selection. **(H)** Venn diagram showing the overlap among genes identified by SVM-RFE, RF, and LASSO analyses.

### Developing a nomogram utilizing mechanical stimulus-associated genes

To construct a diagnostic nomogram integrating the mechanical stimulus-related biomarkers, we first evaluated the individual predictive capacity of each gene via ROC curve analysis (Figures 3A,B). The resulting AUC values indicated that most biomarkers achieved satisfactory diagnostic accuracy (AUC > 0.8), with the exception of THBS1. As shown in Figure 3C, the expression patterns of these 11 genes in HF vs. healthy samples revealed that PPL, STRBP, SLC2A1, PDE2A, and THBS1 were downregulated, whereas PTN, MDK, CASP1, SERPINE2, NPPA, and GADD45A exhibited elevated expression levels in the HF group. Using the rms package in R, we then established a nomogram model incorporating these biomarkers (Figure 3D). The predictive performance of this nomogram was assessed in both the training cohort (GSE141910) and the validation cohort (GSE57338), yielding AUC values of 0.995 and 0.978, respectively (Figures 3E,F). Furthermore, risk scores calculated from the nomogram were substantially higher in HF patients compared to healthy controls (Figures 3G,H). The regression coefficients and corresponding 95% confidence intervals for the 11 genes in the training and independent evaluation cohorts are provided in Supplementary Table S2. Calibration analysis demonstrated the agreement between the predicted and observed probabilities, while DCA indicated the potential clinical net benefit of the model across a range of threshold probabilities in both cohorts (Supplementary Figure S1). Collectively, these findings corroborate the strong predictive capability of the nomogram model for HF diagnosis.

**Figure 3:**
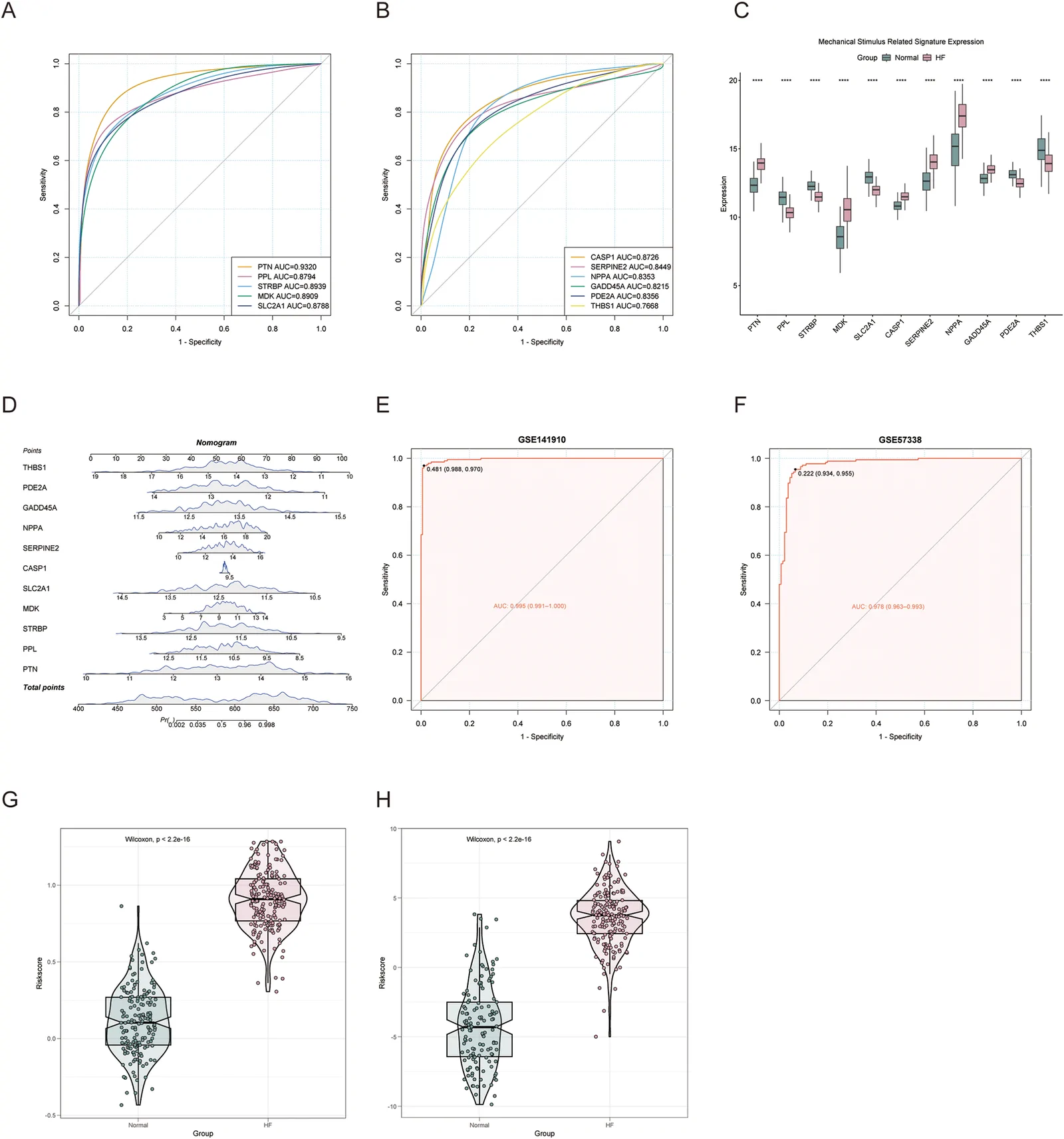
Construction and validation of a diagnostic nomogram based on mechanical stimulus–related biomarkers. **(A,B)** ROC curves evaluating the diagnostic performance of individual biomarkers in GSE141910 cohort. **(C)** Expression levels of the 11 candidate biomarkers in HF and healthy control samples. **(D)** Diagnostic nomogram established using the identified biomarkers. **(E,F)** ROC curves demonstrating the diagnostic performance of the nomogram in the training cohort (GSE141910) and validation cohort (GSE57338). **(G,H)** Comparison of risk scores between HF and healthy samples in the training and validation cohorts.

### Characterization of molecular subtypes linked to mechanical stimuli and their immune microenvironment disparities in HF

We performed consensus clustering on the entire GSE141910 cohort (*n* = 200) to elucidate the link between MSRG expression patterns and HF subtypes. Among the candidate cluster numbers (k = 2–10), k = 2 exhibited the lowest PAC value, indicating the lowest proportion of ambiguous sample assignments (Supplementary Figure S2). In addition, the cluster consensus scores for Cluster A and Cluster B at k = 2 were 0.915 and 0.994, respectively, indicating high within-cluster stability. Together with the consensus matrix and CDF characteristics, these results supported k = 2 as the optimal clustering solution. This suggests the 11 MSRGs successfully divided the patients into two separate groups (Figures 4A,B). Specifically, Cluster A contained 165 individuals, whereas Cluster B consisted of 35. Figure 4C presents a heatmap illustrating the divergent expression signatures of these groups. Subsequently, we analyzed immune infiltration and observed significant variations in the abundance of 22 immune cell subsets across the subtypes (Wilcoxon test, *P* < 0.05; Figure 4D). Notably, Cluster A showed a greater abundance of resting dendritic cells and naive B cells, while Cluster B was dominated by resting mast cells and resting CD4 memory T cells. Regarding immune checkpoints, Cluster A showed markedly upregulated levels of TNFRSF4, LGALS9, ICOSLG, and CD44. Conversely, the expression of TNFSF18, TNFSF4, NRP1, and IDO1 was significantly downregulated in Cluster A relative to Cluster B (Figure 4E).

**Figure 4:**
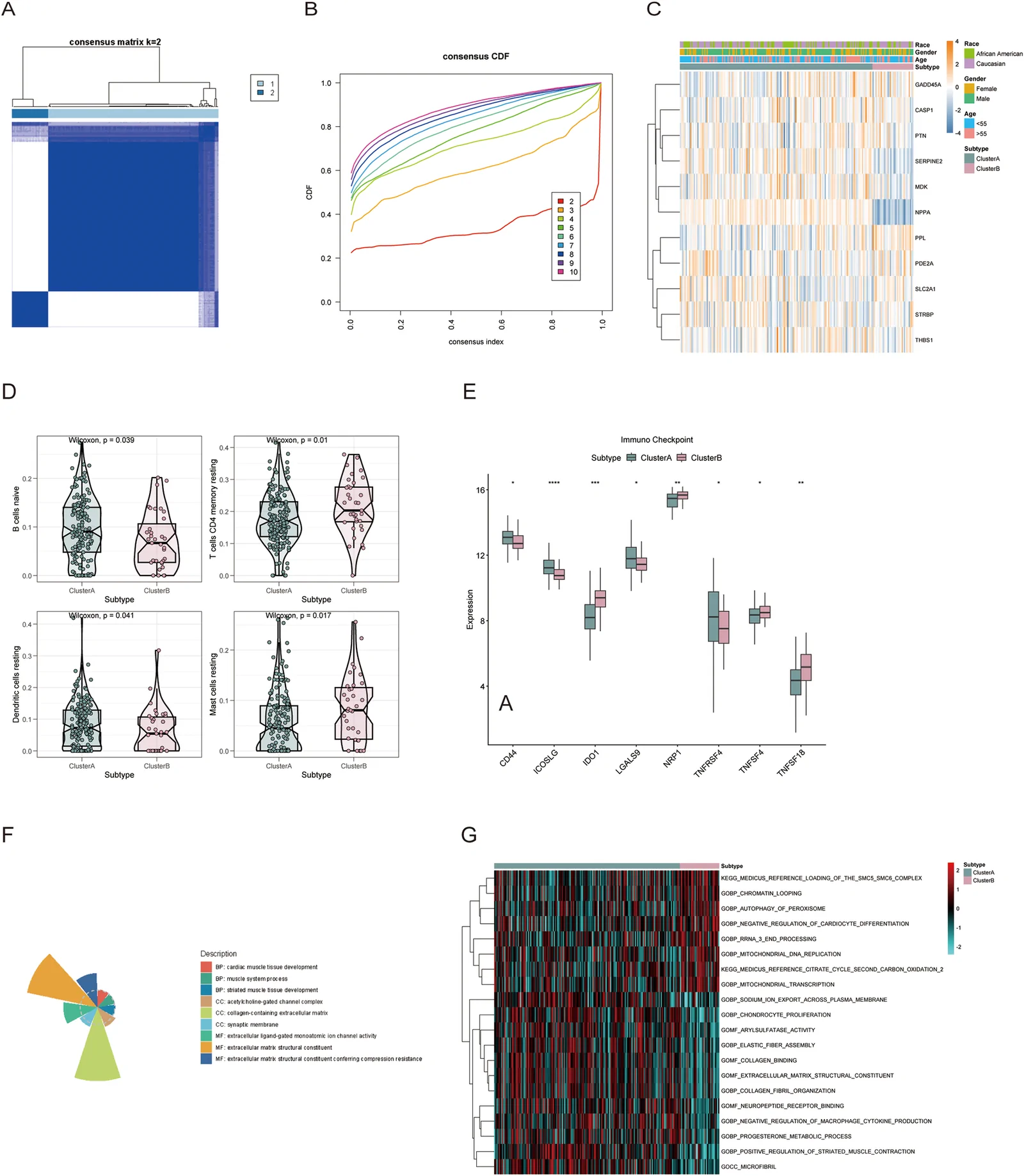
Identification of mechanical stimulus–related molecular subtypes and immune characteristics in HF. **(A)** Consensus clustering matrix showing the optimal classification of HF samples into two molecular subtypes. **(B)** Cumulative distribution function (CDF) curve for determining the optimal cluster number. **(C)** Heatmap showing the expression profiles of the 11 mechanical stimulus–related biomarkers across Cluster A and Cluster B. **(D)** Comparison of infiltrating immune cell populations between the two subtypes. **(E)** Differential expression of immune checkpoint genes between Cluster A and Cluster B. **(F)** GO and KEGG enrichment analyses of subtype-related genes. **(G)** Heatmap illustrating the biological pathway differences between Cluster A and Cluster B.

To uncover the molecular drivers distinguishing these subtypes, we identified 57 subtype-specific genes. GO and KEGG assessments revealed their involvement in processes like striated muscle tissue development, muscle system process, and cardiac muscle tissue development. Furthermore, they were linked to extracellular matrix structural constituent, extracellular ligand-gated monoatomic ion channel activity, collagen-containing extracellular matrix, and acetylcholine-gated channel complex (Figure 4F). Figure 4G highlights the biological divergence between the clusters. Cluster A primarily featured pathways related to autophagy, citrate cycle, chromatin regulation, and mitochondrial function, pointing towards active metabolism and homeostasis. On the other hand, Cluster B was heavily associated with ECM remodeling pathways, such as microfibril formation, elastic fiber assembly, extracellular matrix structural constituent, collagen fibril organization, and collagen binding. This points to a pronounced fibrotic and tissue remodeling state. Ultimately, these results suggest that the two subtypes possess unique biological traits and likely drive HF progression via distinct molecular routes.

### Identification and interaction network analysis of candidate diagnostic biomarkers

To explore potential regulatory relationships involving the candidate diagnostic biomarkers, we constructed a miRNA–mRNA interaction network based on predicted interactions. A total of 558 miRNAs were predicted to interact with the 11 candidate biomarkers (Figure 5). Among these, hsa-let-7e-5p and hsa-miR-15a-5p were predicted to interact with multiple candidate genes, suggesting that they may represent prominent regulatory nodes within the predicted network.

**Figure 5:**
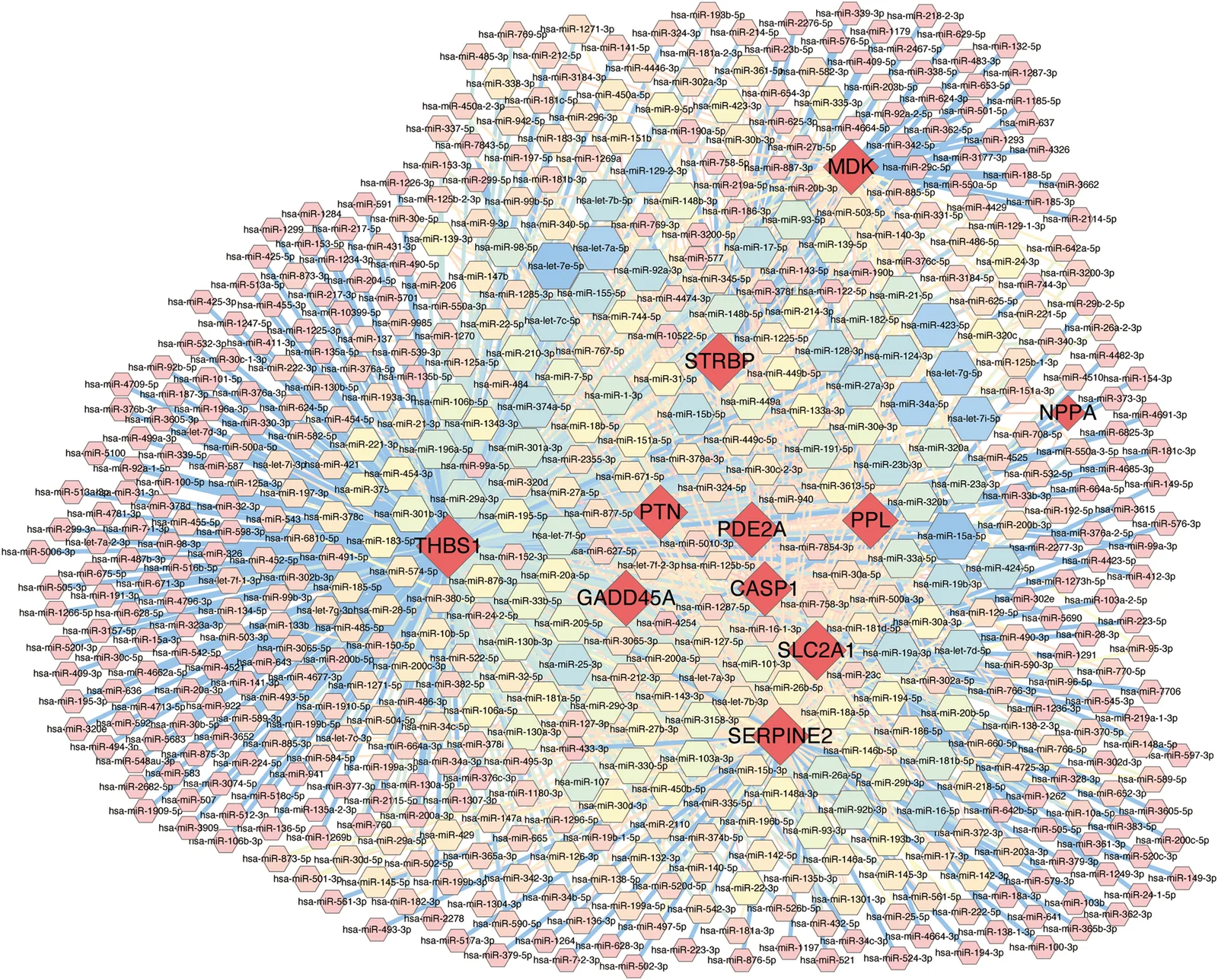
miRNA regulatory network of the candidate diagnostic biomarkers.

### Single-cell transcriptome data analysis

Figure 6A shows the UMAP visualization of the integrated single-cell RNA sequencing dataset, in which 13 distinct cell clusters were identified, indicating substantial cellular heterogeneity. Figure 6B shows the annotation of 11 major cell populations based on the cell-type identities provided with the original dataset, including Fibroblasts, Endothelium, Pericytes, Macrophages, T cells, Smooth Muscle cells, Monocytes, NK cells, Neurons, B cells, and Lymphatic cells. Figure 6C displays the distribution of donor and HF cells across the integrated UMAP space, while Figure 6D illustrates the relative proportions of different cell types between the two groups. Fibroblasts and Endothelial cells were the dominant populations in both groups, whereas HF samples showed increased infiltration of immune-associated cells, particularly Macrophages and T cells, suggesting enhanced inflammatory activation and microenvironment remodeling during HF progression. Figure 6E illustrates the predicted cell–cell communication network, with Fibroblasts and Endothelial cells showing relatively extensive interactions with other cell populations within the cardiac microenvironment. Figure 6F presents the pseudotime trajectory analysis of Fibroblasts, revealing an inferred continuum of transcriptional states and cellular heterogeneity within the fibroblast population.

**Figure 6:**
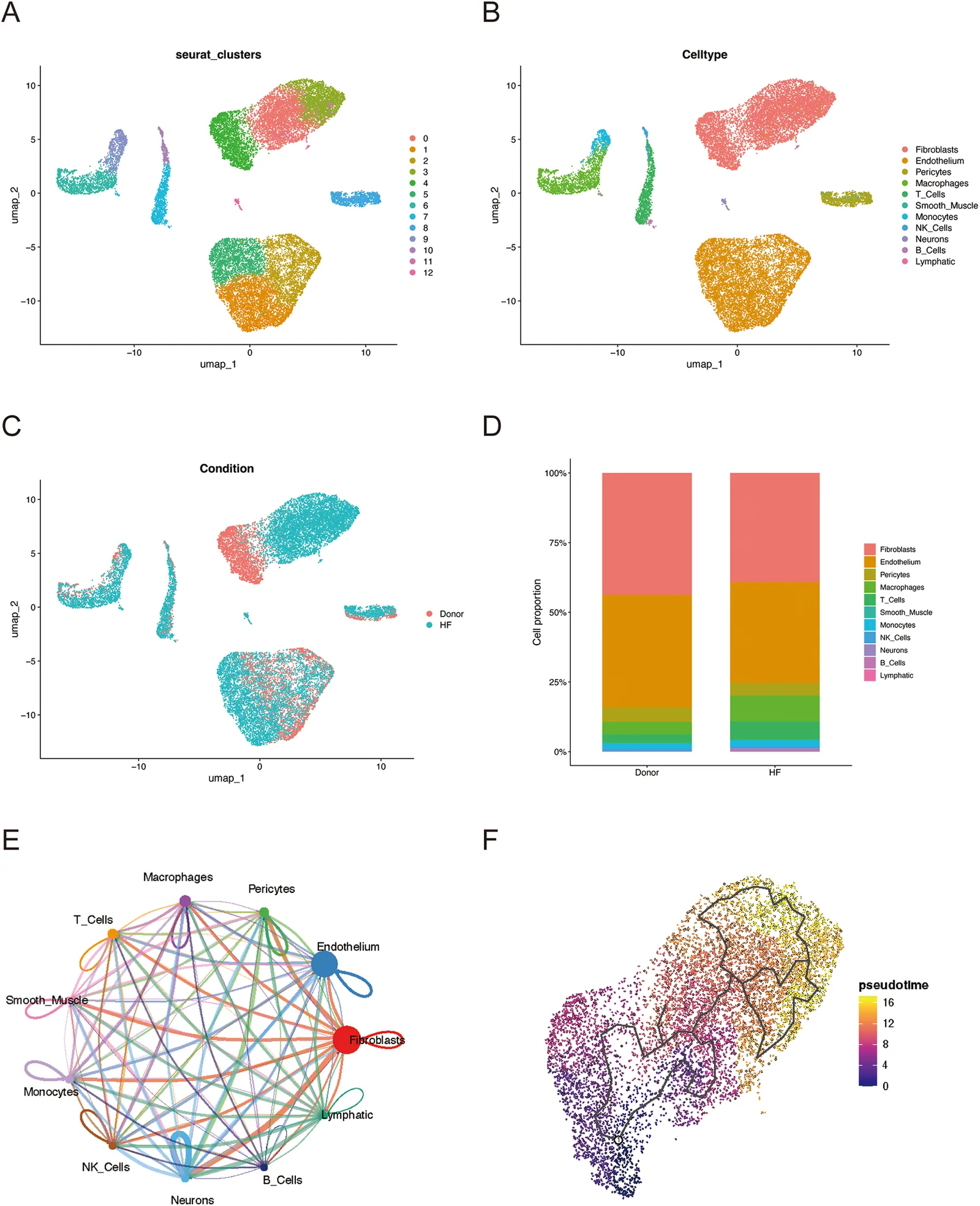
Single-cell transcriptomic analysis of heart failure. **(A)** UMAP visualization of integrated single-cell RNA sequencing data showing 13 distinct cell clusters. **(B)** Cell-type annotation based on canonical marker genes. **(C)** Distribution of donor and HF cells across the UMAP space. **(D)** Relative proportions of different cell types in donor and HF samples. **(E)** Cell–cell communication network showing intercellular interactions among major cell populations. **(F)** Pseudotime trajectory analysis of fibroblasts illustrating an inferred continuum of transcriptional states and cellular heterogeneity within the fibroblast population.

### Mechanical stimulus related markers in microenvironment of HF

Figure 7A illustrates the expression landscape of 11 key MSRGs across distinct cardiac cell populations. Notably, PTN, PPL, STRBP, MDK, SERPINE2, and THBS1 exhibited predominant enrichment in Fibroblasts, suggesting their critical role in fibroblast activation and matrix remodeling. CASP1 was specifically enriched in immune populations, particularly Macrophages and Monocytes, while NPPA expression was observed in specific cellular subsets, though its precise cell-type specificity requires further validation. Endothelial-associated genes such as PDE2A and GADD45A were also distinctly clustered. Figure 7B further confirms these cell type–specific expression patterns via a heatmap, highlighting the distinct molecular signatures separating Fibroblasts, immune cells, and vascular populations. The spatial distribution of these biomarkers on the UMAP embedding is visualized in Figure 7C, validating their specific localization within the tissue microenvironment.

**Figure 7:**
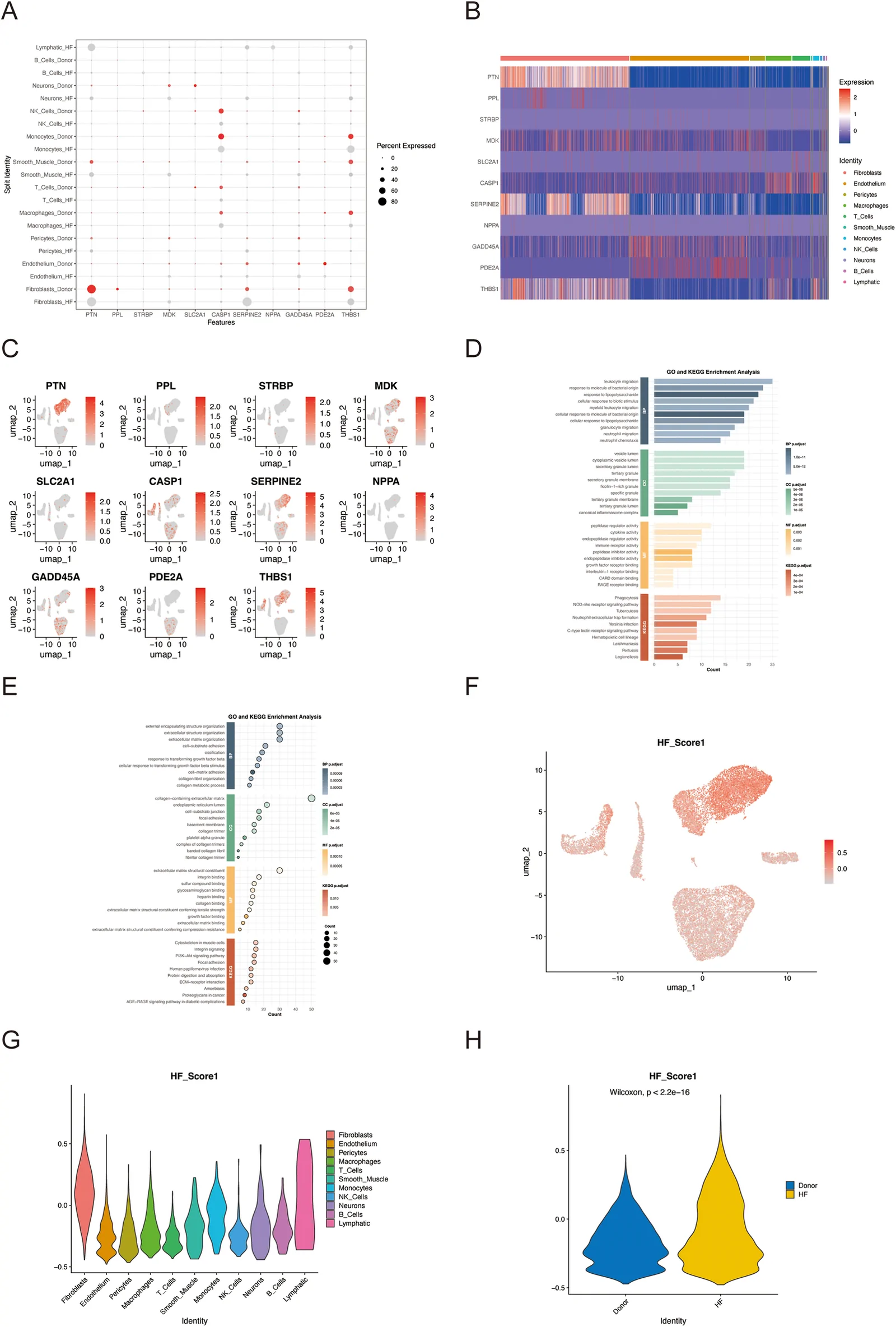
Single-cell characterization and functional analysis of mechanical stimulus–related biomarkers in HF. **(A)** UMAP plot showing the expression distribution of key biomarkers across different cell populations. **(B)** Cell-type–specific expression patterns of the identified biomarkers. **(C)** FeaturePlot illustrating the expression levels of representative biomarkers across cell populations. **(D)** Functional enrichment analysis of CASP1-high myeloid cells. **(E)** Functional enrichment analysis of PTN-high fibroblasts. **(F)** UMAP visualization of the 11-gene signature module score derived from the 11-gene signature. **(G)** Distribution of 11-gene signature module score across different cell types. **(H)** Comparison of 11-gene signature module score between donor and HF samples.

Functional enrichment analysis was performed to elucidate the biological pathways associated with these markers. As shown in Figure 7D, genes associated with the myeloid lineage (e.g., CASP1) were significantly enriched in inflammatory and innate immune pathways. GO analysis highlighted terms such as “leukocyte migration,” “neutrophil chemotaxis,” and “response to lipopolysaccharide,” while KEGG analysis indicated activation of the “NOD-like receptor signaling pathway” and “phagosome,” suggesting that these markers promote inflammasome-mediated inflammatory responses. Conversely, Figure 7E demonstrates that fibroblast-enriched markers (e.g., PTN, SERPINE2) are strongly associated with extracellular matrix (ECM) remodeling. GO terms included “extracellular matrix organization” and “collagen fibril organization,” while KEGG pathways such as “ECM-receptor interaction” and “PI3K-Akt signaling” were significantly enriched, indicating a drive towards fibrosis and tissue stiffening.

Figures 7F–H present the single-cell module score analysis (termed HF_Score1) based on this 11-gene signature. Figure 7F shows that HF_Score1 is highly enriched in Fibroblasts, with moderate expression observed in Monocytes and Lymphatic cells, suggesting a close association with fibroblast activation and inflammatory remodeling. Figure 7G quantitatively confirms that Fibroblasts exhibit the highest module scores among all cell types, followed by Lymphatic and Monocyte populations. Finally, Figure 7H shows the distribution of HF_Score1 in cells derived from Donor and HF samples. HF-derived cells exhibited relatively higher HF_Score1 values; however, this comparison was interpreted descriptively because individual cells were not considered independent biological replicates.

### *In vivo* experimental validation of mechanical stimulus-related biomarkers in a TAC-induced HF model

To verify the clinical utility and pathophysiological significance of the screened mechanosensitive markers in living organisms, quantitative reverse transcription PCR was initially conducted to evaluate their mRNA profiles within myocardial samples obtained from TAC operated rodents alongside sham-operated counterparts. According to Figure 8A, transcriptomic assays exhibited profound aberrations regarding these pivotal transcripts in the pressure-overloaded myocardium. Such mRNA fluctuations closely paralleled the patterns detected in human HF datasets, thereby substantiating the evolutionary preservation of mechanotransduction-associated transcript anomalies under hemodynamic stress. For protein-level verification and functional exploration, three pivotal mechanosensitive regulators (PTN, MDK, and CASP1) were chosen for immunoblotting assays. Immunoblotting outcomes (Figures 8B,C) demonstrated marked upregulation of these three targets in the TAC group vs. the sham cohort. Simultaneously, classical HF indicators along with fibrosis-associated molecules showed substantial accumulation in the surgical model (Figures 8D–I). These data verified the successful generation of the failing heart model while bridging the aberrant expression of PTN, MDK, and CASP1 to adverse myocardial restructuring and fibrogenesis. Additionally, immunohistochemistry (IHC) was utilized to map the spatial localization of these mechanosensitive targets (PTN, MDK, CASP1) together with vital inflammatory mediators (IL-6, IL-1β) within the tissue microenvironment (Figures 8J,K). Histological examinations indicated markedly augmented abundance of the aforementioned trio in TAC-operated subjects relative to controls. Such spatial patterns align perfectly with prior single-cell and enrichment findings, highlighting the macrophage-specific accumulation of CASP1 and its role in triggering inflammasome-driven inflammation. Ultimately, these animal experiments solidly establish that elevated mechanosensitive signatures are fundamentally tied to intensified regional inflammation, immune niche restructuring, and progressive cardiac decline in HF.

**Figure 8:**
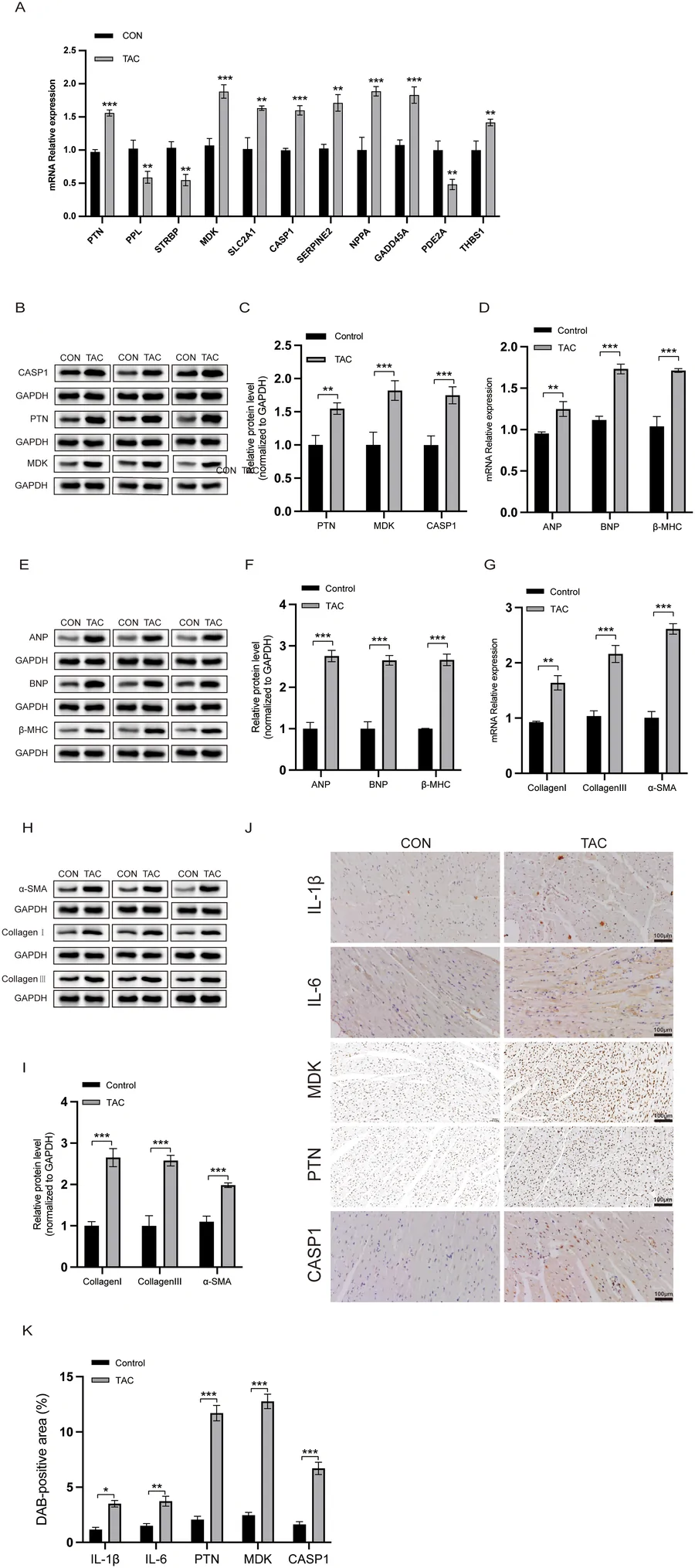
*In vivo* experimental validation of mechanical stimulus-related biomarkers in a TAC-induced heart failure model. **(A)** RT-qPCR showing mRNA levels of 11 core MSRGs in CON and TAC hearts. PTN, MDK, SLC2A1, CASP1, SERPINE2, NPPA, GADD45A, and THBS1 were upregulated, while PPL, STRBP, and PDE2A were downregulated in TAC rats. **(B,C)** Western blot analysis and quantification of CASP1, PTN, and MDK protein levels (normalized to GAPDH). **(D)** RT-qPCR of HF markers ANP, BNP, and β-MHC. **(E,F)** Western blot and quantification of ANP, BNP, and β-MHC. **(G)** RT-qPCR of fibrosis markers Collagen I, Collagen III, and α-SMA. **(H,I)** Western blot and quantification of Collagen I, Collagen III, and α-SMA. **(J)** Immunohistochemical staining showing the protein expression and spatial distribution of IL-1β, IL-6, MDK, PTN, and CASP1 in Control and TAC heart tissues. Protein levels were normalized to GAPDH. **(K)** Quantification of DAB-positive area (%) of IHC staining. Bars represent mean ± SEM. derived from *n* = 3 independent biological replicates (three distinct animals) per group. Significance: *P < 0.05, **P < 0.01, ***P < 0.001.

## Discussion

In the present study, we employed an integrative multi-omics approach combined with machine learning algorithms to systematically identify mechanical stimulus-related diagnostic biomarkers in HF. By analyzing bulk transcriptomic datasets, we identified 11 robust core biomarkers that exhibited exceptional diagnostic performance with AUC values of 0.995 and 0.978 in training and validation cohorts, respectively. However, because the public datasets used in this study are predominantly cross-sectional, the clinical application of this 11-gene panel currently remains at the stage of diagnosis and molecular subtyping, rather than longitudinal prognostic prediction. Furthermore, consensus clustering revealed two distinct molecular subtypes of HF characterized by divergent immune microenvironment profiles and biological pathways. Single-cell RNA sequencing analysis provided unprecedented resolution of the cellular origins and functional roles of these biomarkers, demonstrating their specific enrichment in fibroblasts and immune cells. Most importantly, our *in vivo* TAC model experiments validated the significant upregulation of key markers and established their intrinsic link to pathological cardiac remodeling, inflammation, and fibrosis. Collectively, these findings not only provide novel diagnostic tools but also illuminate the mechanistic cascade connecting mechanical stress to immune microenvironment remodeling in HF.

Among the 11 identified biomarkers, CASP1, MDK, and PTN emerged as central players in a potential pathogenic cascade. Importantly, rather than acting as isolated mechanoreceptors, these biomarkers may represent a “vicious mechanobiological cycle” potentially associated with mechanical stress. Our findings suggest a spatially and functionally coordinated mechanism wherein mechanical overload initiates immune activation and culminates in fibrotic remodeling, which in turn exacerbates wall stress. This recontextualizes these genes not as generic late-stage markers, but as a molecular fingerprint of the mechanical stress-driven pathological cascade.

First, CASP1 functions as the critical mechanotransduction hub that translates hemodynamic overload into innate immune activation. Rather than acting as a passive inflammatory marker, CASP1 serves as the effector protease of the NLRP3 inflammasome, a multiprotein complex exquisitely sensitive to mechanical strain. Recent landmark studies have demonstrated that pressure overload triggers NLRP3 assembly in cardiac macrophages and cardiomyocytes via mechanosensitive pathways, potentially involving mitochondrial ROS generation and subsequent inflammasome activation (34, 35). This mechanical cue leads to CASP1-dependent cleavage and release of pro-inflammatory cytokines, such as IL-1β and IL-18, thereby initiating a sterile inflammatory storm (36, 37). Our multi-omics data robustly validated this paracrine inflammatory niche; scRNA-seq confirmed CASP1-specific enrichment in the macrophage/monocyte lineage, while IHC spatially corroborated its co-localization with downstream IL-1β and IL-6 in TAC hearts. Consequently, CASP1 may act as a represents the primary “mechano-immune checkpoint” that is associated with the initiation of the inflammatory cascade in the overloaded myocardium, effectively bridging physical wall stress to biochemical immune responses (38).

Second, MDK operates as a pivotal paracrine amplifier orchestrating maladaptive intercellular crosstalk within the failing myocardium. Although scRNA-seq localized MDK predominantly to fibroblasts, its pathogenic role extends far beyond structural matrix synthesis. In the mechanostressed microenvironment, fibroblast-derived MDK acts as a potent chemokine-like cytokine that actively recruits and polarizes infiltrating immune cells. Emerging evidence highlights MDK's capacity to drive macrophage polarization and modulate the local immune landscape via receptor-mediated signaling networks, such as the LRP1 axis (39–41). Our consensus clustering revealed that the MDK-high subtype (Cluster B) was intricately linked to distinct immune cell infiltration and profound immune checkpoint exhaustion. Therefore, MDK may serves as a crucial “navigation molecule” that contributes to bridging transient mechanical stress to sustained immune microenvironment remodeling. By facilitating the bidirectional crosstalk between activated fibroblasts and infiltrating macrophages, MDK propagates and amplifies the inflammatory cascade initially triggered by CASP1, ultimately paving the way for irreversible fibrotic progression (42–44).

Finally, PTN emerges as the terminal executor of fibrotic remodeling, effectively closing the vicious mechanobiological loop. Beyond its traditional role in cell survival, PTN secreted by activated fibroblasts directly promotes excessive extracellular matrix (ECM) deposition and myofibroblast differentiation, particularly under pressure-overload conditions (45). Furthermore, PTN intersects with canonical profibrotic cascades, such as the TGF-β/Smad pathway, to lock fibroblasts into a perpetually activated state (46). Crucially, the PTN-driven ECM stiffening fundamentally alters the myocardial mechanical properties, which in turn imposes exacerbated mechanical stress on adjacent cardiomyocytes via integrin-mediated mechanosensing. This “mechano-matrix” positive feedback loop explains why PTN and other cascade markers are universally sustained in late-stage HF regardless of the initial etiology. Ultimately, PTN perpetuates an irreversible vicious cycle of cardiac deterioration, transforming reversible mechanical overload into irreversible fibrotic scarring.

The consensus clustering analysis revealed two distinct molecular subtypes of HF (Cluster A and Cluster B) with fundamentally different biological characteristics and immune microenvironment profiles. Cluster A exhibited enhanced metabolic activity and cellular homeostasis, with enrichment in pathways related to mitochondrial function, chromatin regulation, citrate cycle, and autophagy. In contrast, Cluster B displayed a pronounced fibrotic and tissue remodeling phenotype, with significant enrichment in ECM-related pathways including collagen binding, collagen fibril organization, and extracellular matrix structural constituent. These findings are consistent with emerging concepts of HF heterogeneity and molecular phenotyping (47, 48). The immune infiltration analysis revealed that Cluster B had significantly higher levels of “T cells CD4 memory resting” and “Mast cells resting,” suggesting a chronic inflammatory state. Moreover, the differential expression of immune checkpoints indicates potential immunosuppressive or exhausted immune states that may contribute to disease progression. These findings have important clinical implications, as patients in Cluster B may benefit from anti-fibrotic therapies or immunomodulatory interventions, whereas Cluster A patients might respond better to metabolic modulators. This molecular subtyping framework could enable precision medicine approaches in HF management**.** From a translational perspective, delineating this “mechanical stress-immune-fibrosis” cascade holds clinical implications for diagnosis and molecular subtyping. The 11-gene MSRG panel provides a robust tool for early molecular subtyping and enables the identification of “mechanically-driven” HF phenotypes. However, its current clinical application is limited to diagnosis and molecular subtyping; whether this panel can guide targeted interventions or predict prognosis requires future longitudinal studies.

Our single-cell RNA sequencing analysis provided unprecedented cellular resolution of the mechanical stimulus-related biomarkers and their roles in the cardiac microenvironment. The identification of 13 distinct cell clusters, including fibroblasts, endothelial cells, macrophages, T cells, and smooth muscle cells, revealed substantial cellular heterogeneity in the failing heart. Notably, HF samples showed increased infiltration of immune-associated cells, particularly macrophages and T cells, confirming enhanced inflammatory activation during HF progression. The cell-cell communication network analysis demonstrated that fibroblasts and endothelial cells acted as major interaction hubs, which is consistent with their central roles in ECM remodeling and angiogenesis (49, 50). Furthermore, leveraging single-cell transcriptomics to decipher such complex intercellular crosstalk has been increasingly recognized as a powerful approach in cardiac research. For instance, recent analogous work has successfully utilized single-cell data to unravel intricate myocyte-endothelial communication mechanisms mediated by specific ligand-receptor pairs in HF, further validating the utility of this analytical paradigm in elucidating complex cardiac pathophysiology (51–53). The pseudotime trajectory analysis of fibroblasts revealed transcriptional heterogeneity within this population, though the exact directionality and its link to specific differentiation states require further investigation. Importantly, the cell-type-specific expression patterns of our 11 biomarkers (e.g., CASP1 in macrophages/monocytes, PTN/MDK in fibroblasts) provide mechanistic insights into how different cell populations contribute to HF pathogenesis through distinct molecular programs. These single-cell findings validate and extend our bulk transcriptomic analyses, offering a comprehensive atlas of mechanical stimulus-related gene expression in the cardiac microenvironment. Furthermore, it is essential to acknowledge the confounding effect of cellular proportion changes in bulk transcriptomic analysis. The upregulation of genes like CASP1 and PTN in bulk RNA-seq could result from either increased transcriptional activity per cell or the expanded infiltration of macrophages and fibroblasts in the failing heart. By integrating scRNA-seq and spatial IHC validation, our study effectively disentangled these effects, confirming that the elevated bulk signals genuinely reflect both the activation of specific cellular programs and the pathological expansion of these stromal/immune populations.

The *in vivo* validation in a TAC-induced rat HF model provided crucial biological confirmation of our computational predictions and strengthened the translational relevance of our findings. The TAC model, which mimics pressure-overload mechanical stress observed in human conditions such as hypertension and aortic stenosis, is considered the gold standard for studying mechanically-induced cardiac remodeling (54, 55). The RT-qPCR results revealed significant dysregulation of all 11 core genes in TAC hearts, with expression trends largely mirroring those observed in human HF transcriptomic data, confirming cross-species conservation of mechanical stimulus-related gene dysregulation. The Western blot analysis demonstrated significantly elevated protein levels of PTN, MDK, and CASP1 in TAC hearts, concurrent with robustly increased expression of established HF hallmark proteins and fibrotic markers. These findings not only confirmed successful HF phenotype establishment but also intrinsically linked PTN, MDK, and CASP1 dysregulation to pathological cardiac remodeling and fibrosis. The immunohistochemical staining further revealed that PTN, MDK, and CASP1 expression exhibited overlapping spatial distribution patterns with pro-inflammatory cytokines IL-6 and IL-1β in cardiac tissues, providing spatial evidence that these mechanical stimulus-related biomarkers are associated with exacerbated local inflammation and immune microenvironment remodeling. Collectively, these *in vivo* validations provide supportive expression- and histology-level evidence that is consistent with a potential association between mechanical overload, upregulation of CASP1/MDK/PTN, inflammatory activation, fibrotic remodeling, and cardiac dysfunction, completing the mechanistic cascade from molecular signature to tissue-level pathology (36, 45).

While our multi-omics approach and *in vivo* validation robustly highlight the CASP1-MDK-PTN axis in pressure-overload-induced cardiac remodeling, it is crucial to acknowledge that HF is a highly heterogeneous clinical syndrome. The TAC model primarily simulates mechanical stress, which may not fully capture the distinct molecular profiles driven by other prevalent HF etiologies, such as ischemic or toxicity-induced cardiomyopathy. For instance, recent large-scale epidemiological data underscore that post-myocardial infarction (post-MI) HF remains a highly common complication with distinct temporal dynamics and mortality risks, driven by ischemia-reperfusion injury and subsequent maladaptive remodeling (56). Similarly, chemotherapy-induced HF involves unique molecular mechanisms, predominantly centered around direct cardiomyocyte toxicity, mitochondrial dysfunction, and oxidative stress, which necessitate distinct management strategies (57–59). When cross-comparing our transcriptomic findings with these diverse etiologies, the inflammatory and fibrotic signatures associated with the CASP1-MDK-PTN axis may represent a convergent downstream pathway of cardiac stress; however, their upstream transcriptional triggers likely differ.

Several limitations of this study warrant acknowledgment. First, the public transcriptomic datasets are primarily cross-sectional and derived from different platforms without formal batch correction. This restricts our ability to evaluate longitudinal prognostic value, meaning the 11-gene panel's clinical utility is currently confined to diagnosis and molecular subtyping. Second, the TAC model exclusively utilized male rats, which may not fully capture female-specific pathophysiology or the distinct molecular signatures of other HF etiologies, such as ischemic or toxicity-induced cardiomyopathy. Third, regarding mechanistic validation, the absence of *in vitro* mechanical stretch assays, genetic perturbations, and multiplex immunofluorescence means our data support strong pathophysiological associations rather than definitive causal relationships or true single-cell colocalization. While our IHC provides strong spatial evidence at the tissue level, future studies utilizing immunofluorescence double-staining or multiplex immunohistochemistry are necessary to confirm true single-cell co-localization. Fourth, the scRNA-seq analysis is constrained by a limited number of biological donors and the inherent technical loss of cardiomyocytes during tissue dissociation; furthermore, individual cells were analyzed as nested observations rather than independent replicates to avoid pseudoreplication. Finally, the ceRNA network represents purely computational predictions lacking experimental validation. Future studies incorporating longitudinal real-world tracking, diverse HF models, and rigorous functional experiments are essential to translate these hypotheses into clinical practice.

## Conclusion

In summary, our integrative multi-omics approach identified 11 mechanical stimulus-related diagnostic biomarkers for HF. We propose a mechanistically plausible hypothesis model wherein the CASP1-MDK-PTN axis is potentially associated with immune microenvironment remodeling and fibrotic progression. Coupled with single-cell resolution and *in vivo* validation, we delineated two distinct molecular subtypes, providing a robust framework for early diagnosis and molecular subtyping. While these findings offer novel diagnostic tools, direct causal validation of this cascade will require future *in vitro* mechanical stretch and functional experiments to fully translate these hypotheses into targeted clinical applications.

## Data Availability

The datasets presented in this study can be found in online repositories. The names of the repository/repositories and accession number(s) can be found in the article/Supplementary Material.

## Supplementary material

SUPPLEMENTARY FIGURE S1 Calibration curves and decision curve analysis (DCA) of the nomogram. (A, C) Calibration curves for the GSE57338 and GSE141910 cohorts, respectively. (B, D) DCA for the GSE57338 and GSE141910 cohorts, respectively.

SUPPLEMENTARY FIGURE S2 PAC analysis for determining the optimal cluster number. The x-axis represents the number of clusters (k), and the y-axis represents the PAC score. The lowest PAC value was observed at k = 2, indicating optimal clustering stability.

## References

[1] Ahmed O Behr ER White S Momin A Kaba RAMF. Convergent hybrid ablation versus catheter ablation in patients with persistent atrial fibrillation and heart failure: HALT AF study protocol and rationale for a multicenter randomized controlled trial. J Am Heart Assoc. (2026) 15:e047774. 10.1161/JAHA.125.047774 42216292 PMC13315132

[2] De Fano M Haurum LL Schwarz CR Porcellati F Andersen A. GIP in cardiovascular and kidney disease: from physiology to pharmacology. Diabetes Obes Metab. (2026) 28:6509–32. 10.1111/dom.70888 42209267 PMC13341370

[3] Ono R Chimura M Docherty KF Henderson AD Campbell R Desai AS. Optimal serum potassium concentrations in heart failure: an individual patient data meta-analysis. Eur Heart J. (2026) 47:4480–99. 10.1093/eurheartj/ehag341 42206478 PMC13500161

[4] Abassi Z Khoury EE Karram T Aronson D. Edema formation in congestive heart failure and the underlying mechanisms. Front Cardiovasc Med. (2022) 9:933215. 10.3389/fcvm.2022.933215 36237903 PMC9553007

[5] Chiorescu RM Lazar RDAN Buksa SB Mocan M Blendea D. Biomarkers of volume overload and edema in heart failure with reduced ejection fraction. Front Cardiovasc Med. (2022) 9:910100. 10.3389/fcvm.2022.910100 35783848 PMC9247259

[6] Chowdhury RR Kaur S Gera R. N-terminal pro-b-type natriuretic peptide as a marker of severity of heart failure in children with congenital heart diseases. Pediatr Cardiol. (2023) 44(8):1716–20. 10.1007/s00246-023-03259-x 37563318

[7] Griffith LE Ali MU Andreacchi A Loeb M Kenny M Joshi D. The complexity of examining laboratory-based biological markers associated with mortality in hospitalized patients during early phase of the COVID-19 pandemic: a systematic review and evidence map. PLoS One. (2022) 17(9):e0273578. 10.1371/journal.pone.0273578 36084120 PMC9462773

[8] Yang Z Wang W Wang X Qin Z. Cardiotoxicity of epidermal growth factor receptor 2-targeted drugs for breast cancer. Front Pharmacol. (2021) 12:741451. 10.3389/fphar.2021.741451 34790121 PMC8591078

[9] Zou HY Jiang Z Bian EK Zhou J Li S Yang Y. Nanoencapsulated optical fiber-based PEC microelectrode: highly sensitive and specific detection of NT-proBNP and its implantable performance. Anal Chem. (2025) 97(5):2724–35. 10.1021/acs.analchem.4c04757 39868898

[10] Das P Thandavarayan RA Watanabe K Velayutham R Arumugam S. Right ventricular failure: a comorbidity or a clinical emergency? Heart Fail Rev. (2022) 27(5):1779–93. 10.1007/s10741-021-10192-9 34826024

[11] Stolfo D Collini V Sinagra G. Advanced heart failure in special population: cardiomyopathies and myocarditis. Heart Fail Clin. (2021) 17(4):661–72. 10.1016/j.hfc.2021.05.010 34511213

[12] Zhang X Xia X Zhang Y Liu X Wei X Chen S. Mitochondrial dysfunction and immunoinflammatory remodeling in heart failure: emerging role of conceptualizationthe mtDNA-cGAS/STING axis and therapeutic opportunities. Pharmacol Res. (2026) 227:108195. 10.1016/j.phrs.2026.108195 41997382

[13] Li Q Li C Elnwasany A Sharma G An YA Zhang G. PKM1 exerts critical roles in cardiac remodeling under pressure overload in the heart. Circulation. (2021) 144(9):712–27. 10.1161/CIRCULATIONAHA.121.054885 34102853 PMC8405569

[14] Salido-Medina AB Gil A Expósito V Martínez F Redondo JM Hurlé MA. BMP7-based peptide agonists of BMPR1a protect the left ventricle against pathological remodeling induced by pressure overload. Biomed Pharmacother. (2022) 149:112910. 10.1016/j.biopha.2022.112910 35616049

[15] Schimmel K Ichimura K Reddy S Haddad F Spiekerkoetter E. Cardiac fibrosis in the pressure overloaded left and right ventricle as a therapeutic target. Front Cardiovasc Med. (2022) 9:886553. 10.3389/fcvm.2022.886553 35600469 PMC9120363

[16] Gallo G Rubattu S. Cardiac damage in hypertension: from molecular mechanisms to novel therapeutic approaches. Int J Mol Sci. (2025) 26(12):5610. 10.3390/ijms26125610 40565074 PMC12192565

[17] Liu X Li J Yue Y Li J Wang M Hao L. Mechanisms of mechanical force aggravating periodontitis: a review. Oral Dis. (2024) 30(3):895–902. 10.1111/odi.14566 36989127

[18] Wasim R Singh A Islam A Mohammed S Anwar A Mahmood T. High mobility group box 1 and cardiovascular diseases: study of act and connect. Cardiovasc Toxicol. (2024) 24(11):1268–86. 10.1007/s12012-024-09919-5 39242448

[19] Chen C Wang Z Ding Y Qin Y. Tumor microenvironment-mediated immune evasion in hepatocellular carcinoma. Front Immunol. (2023) 14:1133308. 10.3389/fimmu.2023.1133308 36845131 PMC9950271

[20] Denk D Greten FR. Inflammation: the incubator of the tumor microenvironment. Trends Cancer. (2022) 8(11):901–14. 10.1016/j.trecan.2022.07.002 35907753

[21] Kim M Lee NK Wang C-PJ Lim J Byun MJ Kim T-H. Reprogramming the tumor microenvironment with biotechnology. Biomater Res. (2023) 27(1):5. 10.1186/s40824-023-00343-4 36721212 PMC9890796

[22] Flam E Jang C Murashige D Yang Y Morley MP Jung S. Integrated landscape of cardiac metabolism in end-stage human nonischemic dilated cardiomyopathy. Nat Cardiovasc Res. (2022) 1(9):817–29. 10.1038/s44161-022-00117-6 36776621 PMC9910091

[23] Liu Y Morley M Brandimarto J Hannenhalli S Hu Y Ashley EA. RNA-seq identifies novel myocardial gene expression signatures of heart failure. Genomics. (2015) 105(2):83–9. 10.1016/j.ygeno.2014.12.002 25528681 PMC4684258

[24] Koenig AL Shchukina I Amrute J Andhey PS Zaitsev K Lai L. Single-cell transcriptomics reveals cell-type-specific diversification in human heart failure. Nat Cardiovasc Res. (2022) 1(3):263–80. 10.1038/s44161-022-00028-6 35959412 PMC9364913

[25] Ritchie ME Phipson B Wu D Hu Y Law CW Shi W. Limma powers differential expression analyses for RNA-Sequencing and microarray studies. Nucleic Acids Res. (2015) 43(7):e47. 10.1093/nar/gkv007 25605792 PMC4402510

[26] Zhou Y Zhou B Pache L Chang M Khodabakhshi AH Tanaseichuk O. Metascape provides a biologist-oriented resource for the analysis of systems-level datasets. Nat Commun. (2019) 10(1):1523. 10.1038/s41467-019-09234-6 30944313 PMC6447622

[27] Wang Y Zhuang H Jiang XH Zou RH Wang HY Fan ZN. Unveiling the key genes, environmental toxins, and drug exposures in modulating the severity of ulcerative colitis: a comprehensive analysis. Front Immunol. (2023) 14:1162458. 10.3389/fimmu.2023.1162458 37539055 PMC10394652

[28] Engebretsen S Bohlin J. Statistical predictions with glmnet. Clin Epigenetics. (2019) 11(1):123. 10.1186/s13148-019-0730-1 31443682 PMC6708235

[29] Wilkerson MD Hayes DN. Consensusclusterplus: a class discovery tool with confidence assessments and item tracking. Bioinformatics. (2010) 26(12):1572–3. 10.1093/bioinformatics/btq170 20427518 PMC2881355

[30] Slovin S Carissimo A Panariello F Grimaldi A Bouché V Gambardella G. Single-cell RNA sequencing analysis: a step-by-step overview. Methods Mol Biol. (2021) 2284:343–65. 10.1007/978-1-0716-1307-8_19 33835452

[31] Zheng M Hu Y Liu O Li S Wang Y Li X. Oxidative stress response biomarkers of ovarian cancer based on single-cell and bulk RNA sequencing. Oxidative Med Cell Longev. (2023) 2023:1261039–22. 10.1155/2023/1261039 PMC9897923 36743693

[32] Zhou G Soufan O Ewald J Hancock REW Basu N Xia J. Networkanalyst 3.0: a visual analytics platform for comprehensive gene expression profiling and meta-analysis. Nucleic Acids Res. (2019) 47(W1):W234–41. 10.1093/nar/gkz240 30931480 PMC6602507

[33] Zhou W-W Dai C Liu W-Z Zhang C Zhang Y Yang G-S. Gentianella acuta improves TAC-induced cardiac remodelling by regulating the notch and PI3k/akt/FOXO1/3 pathways. Biomed Pharmacother. (2022) 154:113564. 10.1016/j.biopha.2022.113564 35988427

[34] Higashikuni Y Liu W Numata G Tanaka K Fukuda D Tanaka Y. NLRP3 inflammasome activation through heart-brain interaction initiates cardiac inflammation and hypertrophy during pressure overload. Circulation. (2023) 147(4):338–55. 10.1161/CIRCULATIONAHA.122.060860 36440584

[35] Yan R Sun Y Yang Y Zhang R Jiang Y Meng Y. Mitochondria and NLRP3 inflammasome in cardiac hypertrophy. Mol Cell Biochem. (2024) 479(7):1571–82. 10.1007/s11010-023-04812-1 37589860

[36] Dai F Li X Li X Ding Z Xu R Yin P. Caspase-1 abrogates the salutary effects of hypertrophic preconditioning in pressure overload hearts via IL-1β and IL-18. Front Mol Biosci. (2021) 8:641585. 10.3389/fmolb.2021.641585 33842546 PMC8024560

[37] Tang LQ Wang W Tang QF Wang LL. The molecular mechanism of MiR-26a-5p regulates autophagy and activates NLRP3 inflammasome to mediate cardiomyocyte hypertrophy. BMC Cardiovasc Disord. (2024) 24(1):18. 10.1186/s12872-023-03695-w 38172711 PMC10765805

[38] Chen C Zhang S Sheng M Shao W. NLRP3 inflammasome: a new target for the treatment of CVD and depression comorbidity. Mediat Inflamm. (2025) 2025:4330574. 10.1155/mi/4330574 PMC12585847 41194915

[39] Shi N Chen S Wang D Wu T Zhang N Chen M. MDK promotes m2 macrophage polarization to remodel the tumour microenvironment in clear cell renal cell carcinoma. Sci Rep. (2024) 14(1):18254. 10.1038/s41598-024-69183-z 39107475 PMC11303797

[40] Yuan F Wang Y Yuan L Tang T Ye L Li Y. EGFRvIII-positive glioblastoma contributes to immune escape and malignant progression via the c-fos-MDK-LRP1 axis. Cell Death Dis. (2025) 16(1):453. 10.1038/s41419-025-07771-1 40527884 PMC12174314

[41] Zhang VX Suoangbaji T Tsui Y-M Sze KM-F Lee E Tian L. Repurposing resmetirom suppresses MASH-associated hepatocellular carcinoma, with mechanistic implications of MDK/LRP1-mediated metabolic reprogramming and immunosuppression. Hepatology. (2026) 84:833–50. 10.1097/HEP.0000000000001675 41525571 PMC13480325

[42] Filippou PS Karagiannis GS Constantinidou A. Midkine (MDK) growth factor: a key player in cancer progression and a promising therapeutic target. Oncogene. (2020) 39(10):2040–54. 10.1038/s41388-019-1124-8 31801970

[43] Rasoulizadeh Z Salari AMN Shabgah AG Sargazi G Vakili-Ghartavol R Mohammadpour N. The multifaceted role of midkine in gastrointestinal cancer: from biomarker to treatment. Cancer Treat Res Commun. (2026) 46:101058. 10.1016/j.ctarc.2025.101058 41386217

[44] Shin DH Jo JY Kim SH Choi M Han C Choi BK. Midkine is a potential therapeutic target of tumorigenesis, angiogenesis, and metastasis in non-small cell lung cancer. Cancers (Basel). (2020) 12(9):2402. 10.3390/cancers12092402 32847073 PMC7563676

[45] Sheng K Ran Y Feng X Wang Y Zhou S Guan Y. PTN secreted by cardiac fibroblasts promotes myocardial fibrosis and inflammation of pressure overload-induced hypertrophic cardiomyopathy through the PTN-SDC4 pathway. Life Sci. (2025) 363:123356. 10.1016/j.lfs.2024.123356 39765325

[46] Tang X-C Chen Z-J Chen C-Y Qiu J Huang J-T Tan R-C. ICAM2 loss drives 5-fluorouracil resistance via TGF-β/smad/SP1/PTN-dependent apoptosis evasion and macrophage remodeling in gastric cancer. World J Gastroenterol. (2026) 32(5):115301. 10.3748/wjg.v32.i5.115301 41693979 PMC12897517

[47] Bayes-Genis A Liu PP Lanfear DE De Boer RA González A Thum T. Omics phenotyping in heart failure: the next frontier. Eur Heart J. (2020) 41(36):3477–84. 10.1093/eurheartj/ehaa270 32337540

[48] Lange T Backhaus SJ Schulz A Hashemi D Evertz R Kowallick JT. CMR-based cardiac phenotyping in different forms of heart failure. Int J Cardiovasc Imaging. (2024) 40(7):1585–96. 10.1007/s10554-024-03145-4 38878148 PMC11258094

[49] Tan Y Zhang M Kong Y Zhang F Wang Y Huang Y. Fibroblasts and endothelial cells interplay drives hypertrophic scar formation: insights from *in vitro* and *in vivo* models. Bioeng Transl Med. (2024) 9(2):e10630. 10.1002/btm2.10630 38435816 PMC10905555

[50] Wang S Wang R Yang Y Dong Y Shang P Chen X. Transcriptomic profile reveals cellular composition contribute to hyaline-vascular variant in unicentric castleman disease-associated paraneoplastic pemphigus. Br J Dermatol. (2026) 195:304–15. 10.1093/bjd/ljag153 42008118

[51] Ma Q Li Q Zheng X Pan J. Cellcommunet: an atlas of cell-cell communication networks from single-cell RNA sequencing of human and mouse tissues in normal and disease states. Nucleic Acids Res. (2024) 52(D1):D597–606. 10.1093/nar/gkad906 37850657 PMC10767892

[52] Shao X Lu X Liao J Chen H Fan X. New avenues for systematically inferring cell-cell communication: through single-cell transcriptomics data. Protein Cell. (2020) 11(12):866–80. 10.1007/s13238-020-00727-5 32435978 PMC7719148

[53] Vahid MR Kurlovs AH Andreani T Augé F Olfati-Saber R De Rinaldis E. Disir: fast and robust method to identify ligand-receptor interactions at subunit level from single-cell RNA-Sequencing data. NAR Genom Bioinform. (2023) 5(1):lqad030. 10.1093/nargab/lqad030 36968431 PMC10034587

[54] Clavel MREA Tastet L Pibarot P. Cardiac remodelling in aortic stenosis. Arch Cardiovasc Dis. (2026) 119(1):32–8. 10.1016/j.acvd.2025.11.004 41698772

[55] Ke Q Liu F Tang Y Chen J Hu H Sun X. The protective effect of isosteviol sodium on cardiac function and myocardial remodelling in transverse aortic constriction rat. J Cell Mol Med. (2021) 25(2):1166–77. 10.1111/jcmm.16182 33336505 PMC7812303

[56] Cokic I Chan SF Guan X Nair AR Yang H-J Liu T. Intramyocardial hemorrhage drives fatty degeneration of infarcted myocardium. Nat Commun. (2022) 13(1):6394. 10.1038/s41467-022-33776-x 36302906 PMC9613644

[57] Anastasiou M Oikonomou E Theofilis P Gazouli M Psyrri A Zagouri F. The role of miRNAs in chemotherapy-induced cardiotoxicity. Biomedicines. (2025) 13(10):2331. 10.3390/biomedicines13102331 41153618 PMC12561519

[58] Cheang I Chen Z Yao W Zhang H Li X. Guideline-directed medical therapy in chemotherapy-induced cardiotoxicity and heart failure: current perspectives and practices. Cardiology Plus. (2024) 9:41–8. 10.1097/cp9.0000000000000076

[59] Chen H Zhang L Liu M Li Y Rao P. Multidimensional perspectives on cancer therapy-induced cardiotoxicity: characteristics, mechanisms, biomarkers, psychological stress effects, and treatment progress. Cancer Pathog Ther. (2026) 4(5):362–82. 10.1016/j.cpt.2025.12.003 42597973 PMC13470185

