# Supplementary material for: Integrative multi-omics and machine learning identify mechanical stimulus-related genes and immune microenvironment remodeling in heart failure
Source: Front Cardiovasc Med. 2026 Oct 28;13:1953634. doi: 10.3389/fcvm.2026.1953634 (PMC13751492; doi:10.3389/fcvm.2026.1953634)

Original data of western blotting:
Supplementary figures: Western blotting in the manuscript was performed for three times.The original data of western blotting with three times were shown for Figure8 of the manuscript.

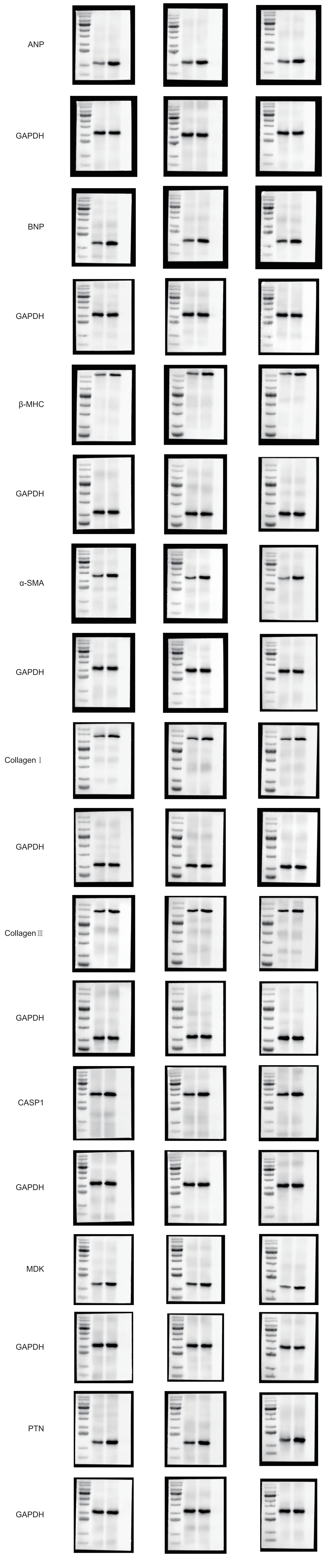

Supplement: Supplementary file 1 [file Datasheet1.pdf]
