# Supplementary material for: Integrative multi-omics and machine learning identify mechanical stimulus-related genes and immune microenvironment remodeling in heart failure
Source: Front Cardiovasc Med. 2026 Oct 28;13:1953634. doi: 10.3389/fcvm.2026.1953634 (PMC13751492; doi:10.3389/fcvm.2026.1953634)
Supplement: Supplementary file 3 [file Table3.docx]

Primer sequences of selected genes.

| **Name** | **Direction** | **Sequence 5′-3′** |
| --- | --- | --- |
| GAPDH | Forward | 5′-CCTCGTCCCGTAGACAAAATG-3′ |
|  | Reverse | 5′-TGAGGTCAATGAAGGGGTCGT-3′ |
| PTN | Forward | 5′-CCAAGCCTCAAGCGGAATCA-3′ |
|  | Reverse | 5′-TTGTCCACAAAAGGTGGCCT-3′ |
| PPL | Forward | 5′-TGACCTTCCTCTGTGTGGAGGACA-3′ |
|  | Reverse | 5′-TTCGCTGTGTGTCTCTCTCCTTGT-3′ |
| STRBP | Forward | 5′-GGCTCTTAGAACCCGCAGTT-3′ |
|  | Reverse | 5′-TCTCTGGAAAGCCCTGAGC-3′ |
| SLC2A1 | Forward | 5′-GAGTGTACTGTGGCCTGACC-3′ |
|  | Reverse | 5′-GTCTAAGCCGAACCTGGG-3′ |
| MDK | Forward | 5′-TAGCGGGAGGGAACGAAGC-3′ |
|  | Reverse | 5′-CTTCGGTGTGTGTGTGTCATCCATCCATC-3′ |
| CASP1 | Forward | 5′-GAAACGCCATGGCTGACAAG-3′ |
|  | Reverse | 5′-CATGATCGCACAGGTCTCGT-3′ |
| SERPINE2 | Forward | 5′-CAAGCGAGGACGGGCTG-3′ |
|  | Reverse | 5′-ACCTGGATCCCTGTGTCAGA-3′ |
| GADD45A | Forward | 5′-CTAAGCAAGAAGCCGGCAAG-3′ |
|  | Reverse | 5′-CCACCGTGTCCATCCTTTCG-3′ |
| PDE2A | Forward | 5′-GGGAGGCAGATGATGAGGAG-3′ |
|  | Reverse | 5′-GGCTCAGCAAAGCATCCTGC-3′ |
| THBS1 | Forward | 5′-AAGACGTCGACGAGTGCAAA-3′ |
|  | Reverse | 5′-CAGGCAGTTGTAGCCAGGAT-3′ |
| ANP | Forward | 5′-AGCGAGCAGACCGATGAAG-3′ |
|  | Reverse | 5′-AGCCCTCAGTTTGCTTTTCA-3′ |
| BNP | Forward | 5′-TTTGGGCAGAAGATAGACCG-3′ |
|  | Reverse | 5′-AGAAGAGCCGCAGGCAGAG-3′ |
| β-MHC | Forward | 5′-TGGAGCTGATGCACCTGTAG-3′ |
|  | Reverse | 5′-ACTTCGTCTCATTGGGGATG-3′ |
| α-SMA | Forward | 5′-CATCAGGAACCTCGAGAAGC-3′ |
|  | Reverse | 5′-TCGGATACTTCAGGGTCAGG-3′ |
| Collagen I | Forward | 5′-GGAAACCTGATGTATGCTTGAT-3′ |
|  | Reverse | 5′-GACTCCTATGACTTCTGCGTCT-3′ |
| Collagen III | Forward | 5′-TGGATTTCAAGATCAACACTGA-3′ |
|  | Reverse | 5′-TCCGGTTTCCATATTACAGAAC-3′ |
